# Incorporating Routine Magnetic Resonance Imaging-based Planning for the Delivery of High-dose-rate Brachytherapy for Prostate Cancer: An Evaluation of Clinical Feasibility and Dosimetric Outcomes

**DOI:** 10.7759/cureus.4085

**Published:** 2019-02-16

**Authors:** Kunal Saigal, Sean All, Peter Potrebko, Nicholas Feranec, Andrew Keller, Mel Lizaso, Chris Warner, Nina Nguyen, Anudh Jain, Matthew Biagioli

**Affiliations:** 1 Radiation Oncology, Florida Hospital, Orlando, USA; 2 Radiation Oncology, University of Central Florida College of Medicine, Orlando, USA; 3 Medical Physics, University of Central Florida College of Medicine, Orlando, USA; 4 Radiology, Florida Hospital, Orlando, USA; 5 Radiation Oncology, University of Pittsburgh Cancer Institute, Pittsburgh, USA

**Keywords:** prostate cancer, prostate, brachytherapy, prostate brachytherapy, high-dose-rate brachytherapy, magnetic resonance imaging, neurovascular bundle

## Abstract

Introduction

To evaluate the implementation and dosimetric outcomes of magnetic resonance imaging (MRI) planning for improved target and normal tissue definition for the treatment of prostate cancer with high-dose-rate brachytherapy (HDRBT).

Methods

From August 2015 to October 2017, 137 unique patients with newly diagnosed localized prostate cancer underwent a total of 174 outpatient brachytherapy procedures using MRI-based treatment planning. Patients receiving brachytherapy as monotherapy underwent two separate procedures while those receiving brachytherapy as a boost after external beam radiation therapy underwent a single procedure. The target volume was defined as the prostate +/- seminal vesicles as clinically appropriate without any additional margin. Pre-treatment dose-volume histogram (DVH) goals to the target were: D90≥95%, V90≥95%, V100≥90%, V150≤30%, V200≤15%. DVH goals to organs-at-risk (OARs): urethra D.01cc ≤115%, bladder D1cc ≤75%, rectum D1cc ≤75%, neurovascular bundle D0.1cc ≤100%, penile bulb D1cc ≤100%. Procedure times were recorded at each step of the procedure, from catheter insertion to removal.

Results

The median target volume was 45.9 cc, the median volume receiving the prescription dose was 53.0 cc, and the median selectivity index was 0.9. The median values for target dosimetry were as follows: D90=99.9%, V90=95.7%, V100=90.1%, V150=28.1%, V200=10.5%. The median values for OAR dosimetry were: urethra D.01cc=114.3%, bladder D1cc=68.3%, rectum D1cc=51.8%, left neurovascular bundle D0.1cc=86.8%, right neurovascular bundle D0.1cc=88.5%, penile bulb D1cc=31.7%. The median time from catheter insertion to end of HDRBT delivery was four hours 14 minutes (range 2:56-9:08); total treatment package time was five hours 32 minutes (range 3:31-9:45).

Conclusion

Routine MRI-based treatment planning is feasible for the delivery of HDRBT for prostate cancer. We met stringent dosimetric criteria despite more objective target and normal tissue definition with MRI imaging. Treatment package time remains reasonable. We have adopted MRI as our standard imaging modality for HDRBT for prostate cancer.

## Introduction

Radiation dose-escalation, through various modalities, has become the standard of care for improving outcomes in patients with newly diagnosed prostate cancer [1-2]. Recently, a phase III randomized controlled trial comparing dose-escalated external beam radiation therapy (DE-EBRT) versus a low-dose-rate (LDR) brachytherapy boost in patients with intermediate and high-risk prostate cancer demonstrated an approximately 20% long-term improvement in biochemical control with a brachytherapy boost when compared to DE-EBRT [3]. This improvement is consistent with multiple prior retrospective series, which suggests that disease control is on a continuum with higher, biologically effective radiation doses [4]. Unfortunately, this improvement in biochemical control did come at the expense of increased toxicity in patients randomized to an LDR brachytherapy boost [5].

An alternative approach to achieving biological dose-escalation is through the delivery of high-dose-rate (HDR) brachytherapy. This technique allows for the delivery of highly conformal brachytherapy via the temporary placement of transperineal brachytherapy catheters through the use of inverse optimization and remote afterloading. Furthermore, due to the growth kinetics of prostate cancer cells exhibiting behavior similar to late-responding normal tissues rather than other, more rapidly dividing tumors, the hypofractionated nature of HDR brachytherapy can offer biologic dose escalation to the target while maximizing the therapeutic ratio of treatment [6]. As such, HDR brachytherapy has become a standard of care treatment modality for prostate cancer, both as monotherapy for low to low-intermediate risk disease and in combination with EBRT in higher risk patients [7].

Contemporaneous with the adoption of HDR brachytherapy as a treatment modality in prostate cancer has been the advancements in multi-parametric magnetic resonance imaging (MRI) of the prostate. The enhanced soft tissue definition offered with high-quality MR imaging of the prostate allows for the identification of higher grade tumors, a bulky tumor volume, as well as the extraprostatic extension of disease. This level of detailed tumor identification has been paradigm shifting in the diagnosis, staging, and management of patients with prostate cancer. From a clinician’s perspective, MR imaging also allows for a better understanding of the borders of the prostate tissue as well as their relationship with surrounding normal structures, including the bladder neck, urethra, anterior fibromuscular stroma, urinary sphincter, and erectile structures [8].

Currently, HDR brachytherapy is planned using either ultrasound (US), computerized tomography (CT), or MRI techniques. Each of these modalities offers unique advantages and disadvantages. Ultrasound-based planning allows for the very efficient delivery of HDR brachytherapy, however, it requires a shielded operating room (OR) for treatment delivery. CT imaging is widely available in radiation therapy departments, however, it offers poor soft tissue definition of prostate anatomy, particularly once brachytherapy catheters are in place. MR imaging offers more objective identification of the prostate tissue as well as OARs, but access to MRI is often limited in radiation therapy departments at present.

At our institution, we are fortunate to have an ambulatory surgery center, MR imaging facility, and radiation therapy department adjacent to one another. This has allowed us to adopt MRI as our primary imaging modality for the treatment planning of HDR brachytherapy. We now report the dosimetric outcomes for MRI-based HDR brachytherapy planning for prostate cancer treatment as well as the feasibility of incorporating this imaging technique into the HDR brachytherapy workflow. Part of this study was presented in the article "Feasibility and dosimetric outcomes of MRI-based planning for delivery of high-dose-rate brachytherapy for prostate cancer; All S, Keller A, Warner C, et al.; Brachytherapy; 2017, 16:S55."

## Materials and methods

This study was conducted after obtaining the approval of a retrospective research protocol through the institutional review board. We identified 137 consecutive patients with newly diagnosed localized prostate cancer who underwent a total of 174 outpatient HDR prostate brachytherapy procedures exclusively using MRI-based planning between August 2015 and October of 2017. Patients receiving brachytherapy as monotherapy underwent two separate brachytherapy procedures. Those receiving brachytherapy as a boost following external beam radiation therapy underwent a single procedure. All brachytherapy procedures were performed by two fellowship-trained radiation oncologists. Forty-one patients underwent placement of SpaceOAR® hydrogel (Augmenix Inc., Massachusetts, USA) prior to external beam radiation therapy and brachytherapy. We excluded patients with a prior history of radiation therapy to the prostate for the present study.

Workflow

All patients underwent the operative insertion of MRI-compatible transperineal brachytherapy catheters (Varian Medical Systems, Palo Alto, California, USA; Liberty Medical, Supply, Inc., Florida, United States) using a disposable brachytherapy template (Best Medical International, Inc., Virginia, USA; Liberty Medical) under general anesthesia using transrectal ultrasound guidance in the lithotomy position. Once catheter positions were finalized, metal obturators were removed to limit catheter migration during patient transfer. Patients were then transported to the department of radiology for MR imaging while remaining anesthetized to limit patient motion. Following MRI acquisition, patients were transferred to the post-anesthesia care unit and awoken from anesthesia. During this interval of post-anesthesia recovery, individual catheter reconstruction, target/normal tissue identification, and treatment planning took place. Once recovered from anesthesia, the patient was transferred to the radiation therapy department and remote-after-loaded HDR brachytherapy was delivered using an Iridium-192 source. Following treatment delivery, the patient was transferred back to the ambulatory surgery center for catheter removal under monitored anesthesia care.

For analysis, we separated the workflow into five main steps as follows: (A-B) from start to end of catheter insertion and template fixation in the operating room, (B-C) MRI, anesthesia recovery, and treatment planning, (C-D) start to end of HDR brachytherapy delivery, (D-E) HDR brachytherapy delivery completion to start of catheter removal in the operating room, and (E-F) start to end of catheter removal. We defined the start of catheter insertion to the completion of treatment as the total time for steps A-D and the total treatment package time as the total time for steps A-F.

Treatment planning

All patients underwent treatment planning using T2-weighted fast spin echo MR images acquired in the axial plane on either a 1.5 Tesla (FOV 240 mm, TR 4000 microseconds, TE 115 microseconds, matrix 320 x 320, voxel resolution 0.5 x 0.5 x 3 mm, 2 NEX) or 3 Tesla (FOV 200 mm, TR 4750 microseconds, TE 121 microseconds, matrix 320 x 320, voxel resolution 0.4 x 0.4 x 3 mm, 2 NEX) General Electric MRI (GE Medical Systems, Chicago, IL, USA). These images were transferred to our brachytherapy treatment planning system (Varian Eclipse (Varian Medical Systems) from August 2017 to December 2016, Elekta Oncentra (Elekta, Stockholm, Sweden) from December 2016 to present). Catheters were then reconstructed using the native axial images as well as sagittal and coronal reconstructions. Great care was taken to identify the signal voids corresponding to each catheter. Of note, as we made the transition from CT-based planning to MRI, we did obtain both image sets on the first 11 patients to gain confidence with MRI-based catheter reconstruction. These patients were excluded from the current analysis, which includes only those who were planned with MRI exclusively.

Following catheter reconstruction, the prostate clinical target volume (CTV), as well as critical structures, including the urethra, bladder, and rectum, were contoured by the treating physician. We also contoured erectile structures, including the neurovascular bundles and penile bulb, and tracked their dosimetry, with the future aim of developing a dose-volume correlation between these structures and post-treatment erectile function. No additional margin was placed upon the CTV for treatment planning purposes. For patients receiving HDR brachytherapy as monotherapy, two fractions of 14 Gy were delivered approximately two to three weeks apart (biologically equivalent dose (BED)=289; equivalent dose in 2 Gy/fr (EQD2)=124, α/β=1.5). For those receiving HDR brachytherapy as a boost, a single fraction of 14-15 Gy was delivered approximately two to four weeks following EBRT, which was delivered to a dose of 45-50.4 Gy delivered via intensity modulated radiation therapy (IMRT) to the prostate and seminal vesicles +/- pelvic lymph nodes (BED=264-275, EQD2=113-118, α/β=1.5).

Dosimetric goals to the target were as follows: V100≥90%, D90 ≥ 95%, V90 ≥ 95%, V150 ≤ 30%, V200 ≤ 15%. Dosimetric goals to organs-at-risk (OAR) were as follows: urethra D.01cc≤115%, bladder D1cc≤75%, rectum D1cc≤ 75%. As mentioned above, dosimetric targets to the neurovascular bundles of D0.1cc≤100% and penile bulb D1cc≤100% were tracked in the DVH evaluation criteria but were not followed at the expense of target coverage.

## Results

The median target volume was 45.9 cc (range: 11.8-129.3 cc). The median number of brachytherapy catheters placed was 15 (range: 13-18). The median volume receiving the prescription dose was 53.0 cc (range: 11.8-141.9 cc), corresponding to a median selectivity index of 0.9 (range 0.6-1.14). The target dosimetric goals and achieved dosimetry are summarized in Table 1. As can be noted, we were generally able to achieve our dosimetric goals without allowing for overly heterogenous brachytherapy treatment plans. OAR dosimetric goals and achieved dosimetry are summarized in Table 2. We achieved our stringent predefined dosimetric goals to the target and normal structures despite the more objective identification of the prostate and normal tissues afforded via MRI imaging. For the purpose of this analysis, we did not separate achieved dosimetry amongst patients with and without SpaceOAR® hydrogel but we are currently evaluating how neurovascular bundle dosimetry is affected by rectal spacing hydrogel placement in a separate study. 

**Table 1:** Dosimetric goals and achieved dosimetry for target volume DVH: dose-volume histogram

DVH Goal	Target	Achieved (median)	Range
V_100_	≥90%	90.1%	71.7-97.5%
D_90_	≥95%	99.9%	71.7-107.6%
V_90_	≥95%	95.7%	80.6-100.7%
V_150_	≤30%	28.1%	17.4-43.4%
V_200_	≤15%	10.5%	4.7-21.0%

**Table 2:** Dosimetric goals and achieved dosimetry to OARs NVB: neurovascular bundle; DVH: dose-volume histogram; OARS: organs-at-risk

DVH Goal	Target	Achieved (median)	Range
Urethra D_.01cc_	≤115%	114.3%	100.8-135.0%
Bladder D_1cc_	≤75%	68.3%	40.6-87.7%
Rectum D_1cc_	≤75%	51.8%	17.3-87.7%
Penile Bulb D_1cc_	≤100%	31.7%	0-82.6%
Left NVB D_0.1cc_	≤100%	86.8%	63.4-246.6%
Right NVB D_0.1cc_	≤100%	88.5%	59.3-376.0%

**Figure 1:**
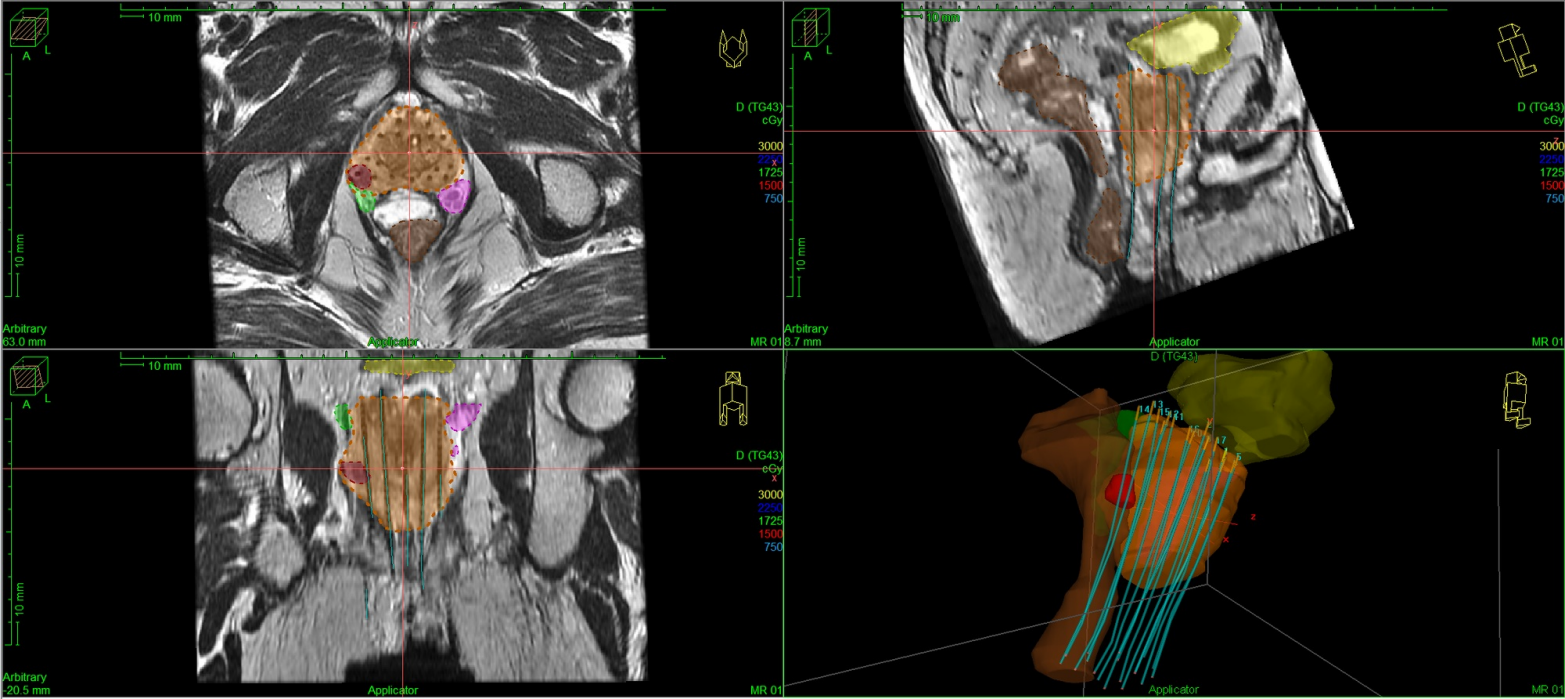
Example of prostate contour and catheter reconstruction Upper left: axial plane; upper right: sagittal plane; lower left: coronal plane; lower right: 3D reconstruction of catheters, target, and organs at risk. Note: contours of prostate clinical target volume (CTV) (orange), gross tumor volume (GTV) (red), neurovascular bundles (green, magenta), bladder (yellow), rectum (brown), and placement of SpaceOAR® hydrogel (T2 hyperintense material). SpaceOAR®: Augmenix Inc., MA, US

**Figure 2:**
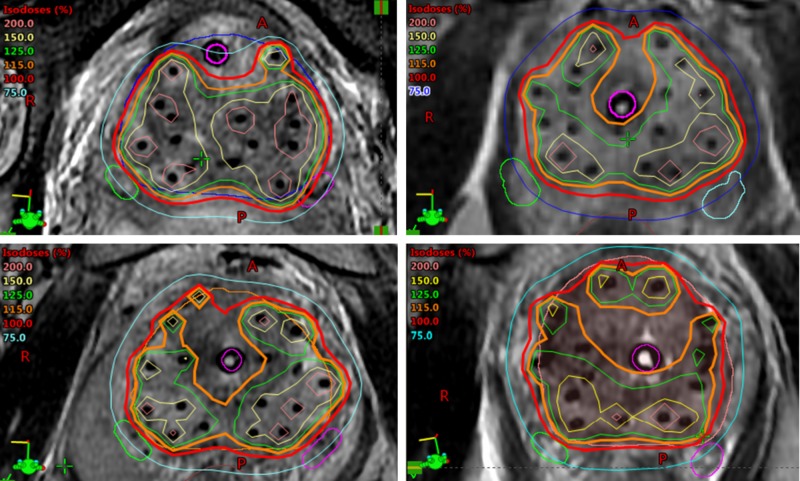
Examples of HDR brachytherapy dosimetry Four (upper left, upper right, lower left, lower right) representative patient examples of MRI-based HDR brachytherapy dosimetry in the axial plane. Note contours of neurovascular bundles at posterior-lateral aspects of prostate gland. MRI: magnetic resonance imaging; HDR: high dose rate

As mentioned previously, we tracked dosimetry to the erectile structures, including the neurovascular bundles and penile bulb, which are more readily identifiable using MR imaging. An example of catheter reconstruction as well as prostate and OAR delineation is shown in Figure 1. Examples of achieved dosimetry are shown in Figure 2.

Median follow-up for the entire cohort was 5.1 months. This was defined from the date of treatment completion to the date of the last follow-up. Median pre-treatment prostate-specific antigen (PSA) was 7.3 ng/mL (range: 1.82-84 ng/mL). Median post-treatment PSA was 0.59 ng/mL (range: 0.01-22.1 ng/mL). We also evaluated the feasibility of integrating MRI imaging into the HDR brachytherapy workflow. Results for various phases of the workflow are summarized in Table 3. Incorporation of MR imaging did not affect our ability to deliver HDR prostate brachytherapy in the outpatient setting, nor did it affect our ability to perform more than one implant per day.

**Table 3:** Evaluation of brachytherapy workflow MRI: magnetic resonance imaging; HDR-BT: high dose rate brachytherapy

Procedure Step	Median Time	Range	Standard Deviation (min)
Catheter insertion (A-B)	0:29	0:10-1:33	21.01
MRI/Recovery/Treatment Planning (B-C)	3:24	2:01-8:27	45.51
HDR BT Delivery (C-D)	0:20	0:08-0:57	6.44
End of HDR BT to Catheter Removal (D-E)	0:56	0:06-4:19	51.83
Catheter Removal (E-F)	0:07	0:01-0:31	4.92
Catheter Insertion →Treatment End (A-D)	4:14	2:56-9:08	48.7
Total Treatment Package (A-F)	5:32	3:31-9:45	69.52

## Discussion

Prostate brachytherapy allows for radiation dose-escalation to the prostate gland beyond what is readily achievable using even the most conformal EBRT techniques [9]. This benefit of further dose-escalation is supported by post-EBRT biopsy data from the Memorial Sloan Kettering group, which demonstrates a 15%-20% risk of local failure following DE-EBRT [10]. Local failure appears to portend the ultimate distant progression of disease both in this series as well as in the androgen suppression combined with elective nodal and dose-escalated radiation therapy (ASCENDE-RT) trial [3]. However, the benefits of dose escalation must be weighed against the risk of increased toxicity with brachytherapy, which is likely due to the delivery of higher radiation doses to normal structures. This was demonstrated in the ASCENDE-RT trial, where increased genitourinary (GU) toxicity was largely secondary to the development of urethral strictures, which the authors suggest is due to high radiation doses delivered to the urinary sphincter and membranous urethra through their EBRT and LDR brachytherapy techniques [5].

One of the challenges of prostate cancer radiation treatment planning is that critical structures are located both within the target, in the case of the prostatic urethra, as well as in intimate proximity to the anatomical boundaries of the target (bladder neck, urinary sphincter, and erectile tissues). In order to maximize the therapeutic ratio of radiation therapy, careful delineation of the prostate as well as these OARs is critical and is available through the incorporation of MR imaging into the brachytherapy workflow. Integration of MRI also allows for further dose escalation to dominant intraprostatic lesions (DILs), which can further improve outcomes without the need to deliver higher radiation doses to the entire prostate gland [11]. Lastly, the use of MR imaging for treatment planning also allows for the clear visualization of commercially available hydrogel spacers, which can further improve rectal dosimetry [12].

HDR brachytherapy offers a unique advantage over LDR because there is the opportunity to perform real-time MRI-based treatment planning, without the inherent need for image co-registration with ultrasound imaging, which can be an additional source of error [11]. Currently, CT imaging is commonly used for HDR brachytherapy treatment planning. This imaging modality offers limited objective visualization of the prostate gland once the brachytherapy catheters have been placed. The result of this is the physician performing some degree of cognitive fusion of pretreatment MR images/intraoperative ultrasound images and treatment-planning CT images. This introduces a potential inaccuracy in the contouring of the target whereby the physician has the tendency to “contour the catheters,” which can artificially inflate dosimetric outcomes. Using MRI-based planning, the anatomic borders of the prostate can be clearly visualized even with brachytherapy catheters in place, removing much of this subjectivity (Figure 1). Nevertheless, we were able to meet the dosimetric criteria for target coverage and OARs, which have been defined by previous studies using CT-based planning [13]. Furthermore, we are able to visualize OARs, which are not readily visible on CT imaging, such as the neurovascular bundles/plexus, which closely approximate the prostatic tissue but have proven to be critical to erectile preservation in the surgical literature [14-15]. When clinically appropriate (no extracapsular extension of the tumor into the neurovascular bundle), we currently use a modest dose constraint to essentially avoid a hotspot within the neurovascular bundles. As our data matures, we will correlate post-treatment erectile function with the neurovascular bundle and penile bulb dosimetry to develop dose-volume constraints for these structures using MRI-based HDR brachytherapy. This will be the subject of a future investigation.

The integration of MRI-based planning into HDR brachytherapy has largely been limited by access to MR imaging in the radiation therapy workflow. Working with our colleagues in the radiology and anesthesiology departments, we created a protocol to acquire a single high-quality MRI series (approximate acquisition time: 12 min) under general anesthesia, which allows us to maintain a very reasonable treatment package for outpatient HDR brachytherapy and consistent with the experience at the University of Toronto using CT-based planning [13]. Therefore, we propose that the incorporation of MRI is feasible from a workflow and patient throughput perspective. Our technique of essentially replacing CT with MRI in the HDR brachytherapy workflow is translatable to more radiation therapy centers than the previously described method of intraoperative MR imaging by Menard and colleagues, which requires considerable investment in infrastructure and an average anesthesia time of four hours [16]. Their work was, to our knowledge, the first using exclusively an MRI-based HDR brachytherapy workflow; ours, however, is novel in that our workflow does not require an intraoperative MRI unit or a shielded operating room for treatment delivery.

The integration of MRI has also been limited by concerns regarding the accuracy of catheter reconstruction. This is due to limitations in MR image slice thickness, as well as uncertainty regarding catheter pathways and tips due to resultant signal voids noted on MRI. While the typical image slice thickness is 1-1.5mm for CT-based brachytherapy planning, the optimal slice thickness for MRI acquisition was 3 mm in our experience, with a further reduction in slice thickness, leading to an unfavorable signal-to-noise ratio and degradation of MR image quality. This does, however, lead to some uncertainty in catheter reconstruction in the superior-inferior plane. However, by acquiring MR images under general anesthesia, there is limited patient motion, which considerably improves the quality of obtained images. In our early experience, we did acquire both CT and MRI image sets until our medical physics team felt certain that accurate catheter reconstruction could be performed using MRI exclusively. We are currently reviewing these cases in a systematic fashion to evaluate any variances in catheter reconstruction and the dosimetric impact of any such translations and intend to publish this work separately. In a similar analysis by Rylander et al., relatively small variances were noted in target and OAR dosimetry when comparing catheter reconstruction and target/OAR delineation across CT, MRI, and US imaging [17]. Furthermore, we and others are evaluating the use of MRI-compatible catheter visualization strategies, which would help overcome any uncertainty in signal voids created by the catheters [18].

Another limitation of MRI-based planning is the inability to re-image patients to evaluate for catheter migration prior to treatment delivery. Even with the delivery of single-fraction brachytherapy, the University of Toronto group has demonstrated a mean internal catheter displacement of 11 mm using cone-beam CT imaging prior to treatment delivery [19]. Given the highly optimized delivery of HDR brachytherapy, small changes in catheter position may have significant impacts on the dosimetric quality of the treatment plan. We do use flexible, MRI-compatible, brachytherapy catheters, and remove the needle obturators prior to repositioning the patient from the lithotomy position into the supine position. With this technique, as well as applying mild superior pressure on the catheters as the patient’s legs are repositioned, we propose that the flexible catheters are maintained in place by the dense prostatic stromal tissue, allowing for minimal migration. Another concern with all modalities of brachytherapy delivery is the changes in prostate anatomy and glandular volume due to catheter-induced trauma and the resultant edema. A recent publication using the aforementioned intraoperative MRI technique revealed an approximate 9% change in glandular volume, with small resultant changes in planning target volume (PTV) coverage but without significant negative impact on OAR dosimetry when reacquiring images prior to treatment delivery. In their study, catheter migration was quite minimal, although patients were maintained in the lithotomy position during the entire procedure, suggesting that the repositioning of patients may be a source of catheter movement [20]. Despite these uncertainties, the clinical results and toxicity of HDR brachytherapy for prostate cancer remain quite favorable [21].

Additional limitations of this study include its retrospective nature, single-institution experience, and limited duration of follow-up. With further maturation of our data, we expect to publish detailed clinical, toxicity, and quality of life endpoints.

## Conclusions

MR imaging can be successfully integrated into the HDR brachytherapy workflow while maintaining both the efficiency of outpatient procedures and the dosimetric quality of brachytherapy delivery while providing more objective identification of the target volume as well as organs-at-risk. Consequently, we have adopted MRI as our standard imaging modality for HDR prostate brachytherapy at our institution. We propose that our work may lay the foundation for the use of MRI-based planning to become widespread, as the ability to accurately visualize the target is critical to optimizing the therapeutic ratio of brachytherapy. Our future work will focus on correlating post-treatment erectile and urinary function to develop dose-volume relationships to the erectile and urinary structures, respectively.
